# Rebamipide protects against glaucoma eyedrop-induced ocular surface disorders in rabbits

**DOI:** 10.1371/journal.pone.0186714

**Published:** 2017-10-19

**Authors:** Ichiro Kawaguchi, Akira Kobayashi, Tomomi Higashide, Yasuhiro Takeji, Kazushi Sakurai, Chiaki Kawaguchi, Kazuhisa Sugiyama

**Affiliations:** 1 Department of Ophthalmology, Kanazawa University Graduate School of Medical Science, Kanazawa, Ishikawa, Japan; 2 Otsuka Pharmaceutical Co., Ltd, Ako Research Institute, Ako, Hyogo, Japan; Xiamen University, CHINA

## Abstract

**Purpose:**

This study aimed to determine if rebamipide eyedrops can improve ocular surface damage caused by the use of glaucoma eyedrops.

**Methods:**

Female Kbl:Dutch rabbits were used to evaluate glaucoma eyedrop-induced ocular surface damage; one eye of each rabbit was untreated and the other was administered glaucoma eyedrops for 30 days. To evaluate the effects of rebamipide on ocular surface damage, one eye of each rabbit was administered vehicle-treated glaucoma eyedrops and the other was administered rebamipide-treated glaucoma eyedrops for 30 days. Corneal and conjunctival epithelial damage was evaluated using fluorescein and rose bengal staining, respectively. Conjunctival inflammation was observed by light microscopy with hematoxylin-eosin staining. Dark cells (in which the corneal microvilli were damaged) were analyzed by scanning electron microscopy.

**Results:**

There were no significant differences in fluorescein staining between the untreated and glaucoma eyedrop-treated groups; however, rose bengal staining and the number of inflammatory cells in the conjunctiva significantly increased after glaucoma eyedrop treatment. There was a four-fold increase in the number of dark cells in the glaucoma eyedrop-treated group compared to untreated. In contrast, in the conjunctiva of the rebamipide-treated glaucoma eyedrop group, rose bengal staining scores, the number of inflammatory cells, and the number of dark cells were decreased compared to the vehicle-treated glaucoma eyedrop group.

**Conclusions:**

Results from our *in vivo* rabbit study demonstrated that short-term use of glaucoma eyedrops induces corneal epithelium disorders at the cellular level, but that simultaneous use of rebamipide has the potential to protect and repair the ocular surface.

## Introduction

Glaucoma eyedrops are the first treatment of choice to control intraocular pressure (IOP) in patients suffering from glaucoma. Recent advancements in glaucoma eyedrops have enabled a more effective reduction in IOP, avoiding or reducing the potential need for early surgical intervention. While the treatment goals for glaucoma can be successfully achieved through the use of glaucoma eyedrops, many patients complain of dry eye symptoms [1], likely attributed to adverse effects caused by the eyedrops. It is also known that the long-term use of glaucoma eyedrops can cause corneal and conjunctival disorders; these include reductions in tear break-up time and basal tear secretion [1, 2], decreased goblet cell density [3, 4], inflammation of the corneal and conjunctival epithelium [5, 6], and increased conjunctival epithelial cell apoptosis [7]. In addition, a correlation between the number of daily glaucoma eyedrops and the severity of superficial punctate keratitis has been reported [8, 9]. The main cause of these ocular surface disorders in patients with glaucoma has been reported to be the preservative benzalkonium chloride (BAK) [10–12], which is commonly used as a preservative in glaucoma eyedrops. When these symptoms occur, ideally, the use of eyedrops should be stopped; however, glaucoma patients may suffer a worsening of visual field defects caused by elevated IOP. Thus, it is necessary to identify methods that can prevent or improve ocular surface damage while continuing treatment with glaucoma eyedrops.

Rebamipide (CAS 11911-87-6) was initially developed in Japan as an oral anti-gastritis or anti-ulcer drug, and has been widely used in Asia. Rebamipide’s mechanism of action in the prevention and treatment of gastritis and/or gastric ulcer is thought to be an increase in mucus secretion and an anti-inflammatory effect for the protection and repair of the gastric mucosa [13–15]. Rebamipide has also been developed as an eyedrop for its mucus-enhancing effects [16] and is prescribed in Japan as a dry eye treatment [17, 18]. We therefore hypothesize that rebamipide eyedrops may re-stabilize the tear film that is destabilized by use of glaucoma eyedrops, and may improve ocular surface symptoms. Utilizing an *in vivo* rabbit model, this study aimed to identify if rebamipide eyedrops can improve damage to the ocular surface resulting from the use of glaucoma eyedrops.

## Materials and methods

### Animals and treatments

The present study was approved by the Institutional Animal Care and Use Committee of Otsuka Pharmaceutical Co., Ltd. (approval number 12–035) and followed the Association for Research in Vision and Ophthalmology (ARVO) statement on the use of animals in ophthalmic and vision research. All experiments were carried out in accordance with the Otsuka Pharmaceutical Co., Ltd. Guidelines on Animal Experiments. Female Kbl:Dutch rabbits (16–19 weeks old, 1.5–2.0 kg; Kitayama Labes, Nagano, Japan) were used. The rabbits were placed in cages (one rabbit per cage) and housed under standard conditions (12-hour light/dark cycle); animals were fed a standard laboratory diet with free access to water throughout the study. Thirty-six rabbits (72 eyes) were used.

In this study, we used commercially available ophthalmic solutions, 2% rebamipide (Mucosta^®^ ophthalmic suspension UD2%; Otsuka Pharmaceutical Co., Ltd., Tokushima, Japan), 0.005% latanoprost (Xalatan^®^; Pfizer, NY, USA), and a fixed combination of 0.5% timolol maleate and 1% dorzolamide (FCTD, Cosopt^®^ [Japanese formulation]; Merck, NJ, USA). In this experiment, all eyes treated with glaucoma eyedrops received both glaucoma eyedrops (latanoprost and FCTD).

First, to evaluate glaucoma eyedrop-induced ocular surface disorders, 16 rabbits (32 eyes) were used: 5 rabbits were used for the evaluation of corneal and conjunctival damage, 5 rabbits were used for the evaluation of corneal microstructure by SEM, and 6 rabbits were used to evaluate regional changes in corneal microstructure. The right eye of each rabbit was untreated and the left eye was administered both glaucoma eyedrops (latanoprost and FCTD) for 30 days.

Second, to evaluate the effects of rebamipide on ocular surface disorders, 20 rabbits (40 eyes) were used: 10 rabbits were used to evaluate corneal and conjunctival damage, and 10 rabbits were used for the evaluation of corneal microstructure. The right eyes were administered vehicle-treated glaucoma eyedrops (latanoprost and FCTD) and the left eyes were administered rebamipide-treated glaucoma eyedrops (latanoprost and FCTD) for 30 days.

For the glaucoma eyedrop (latanoprost/FCTD)-treated group, 50 μL of latanoprost and FCTD were administered topically, once daily for latanoprost (9 AM) and twice daily for FCTD (9 AM and 6 PM) for 30 days. For the rebamipide or vehicle-treated groups, 50 μL of rebamipide or vehicle were administered topically four times daily (9 AM, 12 AM, 3 PM, and 6 PM) for 30 days. All eyedrops were given within a 10-minute interval. In humans, a 5-minute period is typical for the clinical administration of eyedrops. However, to avoid dilution and to achieve maximum effects from each of the eyedrops, we used a 10-minute interval in the animal experiments. For eyes in the rebamipide or vehicle-treated groups, rebamipide or vehicle were given first, followed by the glaucoma eyedrops.

The following methods were used to perform sequential fluorescein staining and rose bengal staining. At day 30, all rabbits were euthanized with an intravenous overdose injection of pentobarbital sodium (Kyoritsu Seiyaku, Tokyo, Japan). Conjunctival inflammation was evaluated by light microscopy. Histomorphological changes to the cornea were observed by scanning electron microscopy (SEM; JSM-6340F, JEOL Ltd., Tokyo, Japan).

### Evaluation of corneal and conjunctival epithelial damage

Corneal and conjunctival epithelial damage was evaluated based on fluorescein and rose bengal staining, respectively. For the evaluation of corneal epithelial damage, 1 μL of 1% fluorescein sodium was instilled into the conjunctival sac. The staining was examined using a scanning laser ophthalmoscope (F-10, NIDEK, Aichi, Japan). Three corneal areas were evaluated (upper, middle and lower), and the regions of positive corneal staining were scored as 0 (absent), 1 (mild), 2 (moderate) or 3 (diffuse loss of epithelium) [19]. The evaluation and scoring of the specimens was done by a single investigator. Total scores ranged from 0 to 9 points. For the evaluation of conjunctival epithelial damage, 1 μL of 1% rose bengal was instilled into the conjunctival sac. The staining was examined under a slit-lamp microscope (Topcon, Tokyo, Japan). Rose bengal staining was scored as 0 (absent), 1 (mild), 2 (moderate) or 3 (diffuse staining) in four regions of the conjunctiva (upper and lower parts of the palpebral, and upper and lower parts of the bulbar conjunctiva) [16]. Total scores ranged from 0 to 12 points.

### Measurement of inflammatory cells in the conjunctival epithelium

At day 30, rabbits were euthanized with an overdose of anesthesia. Eyes were isolated and fixed in 10% formalin. The specimens were embedded in paraffin and sectioned at 4 μm thickness. Histological changes were observed by hematoxylin-eosin staining. A vertical incision around the center of the eye was made to create a resected segment. Two unconnected segments from each specimen were selected, and the number of inflammatory cells (neutrophils and eosinophils) were counted from the upper and lower conjunctiva of both specimens. Two nonadjacent sections were randomly selected for counting, and the average was calculated. The quantification of inflammatory cells in the conjunctival epithelium was performed in a masked fashion.

### Evaluation of the corneal surface by scanning electron microscopy

**Fig 1:**
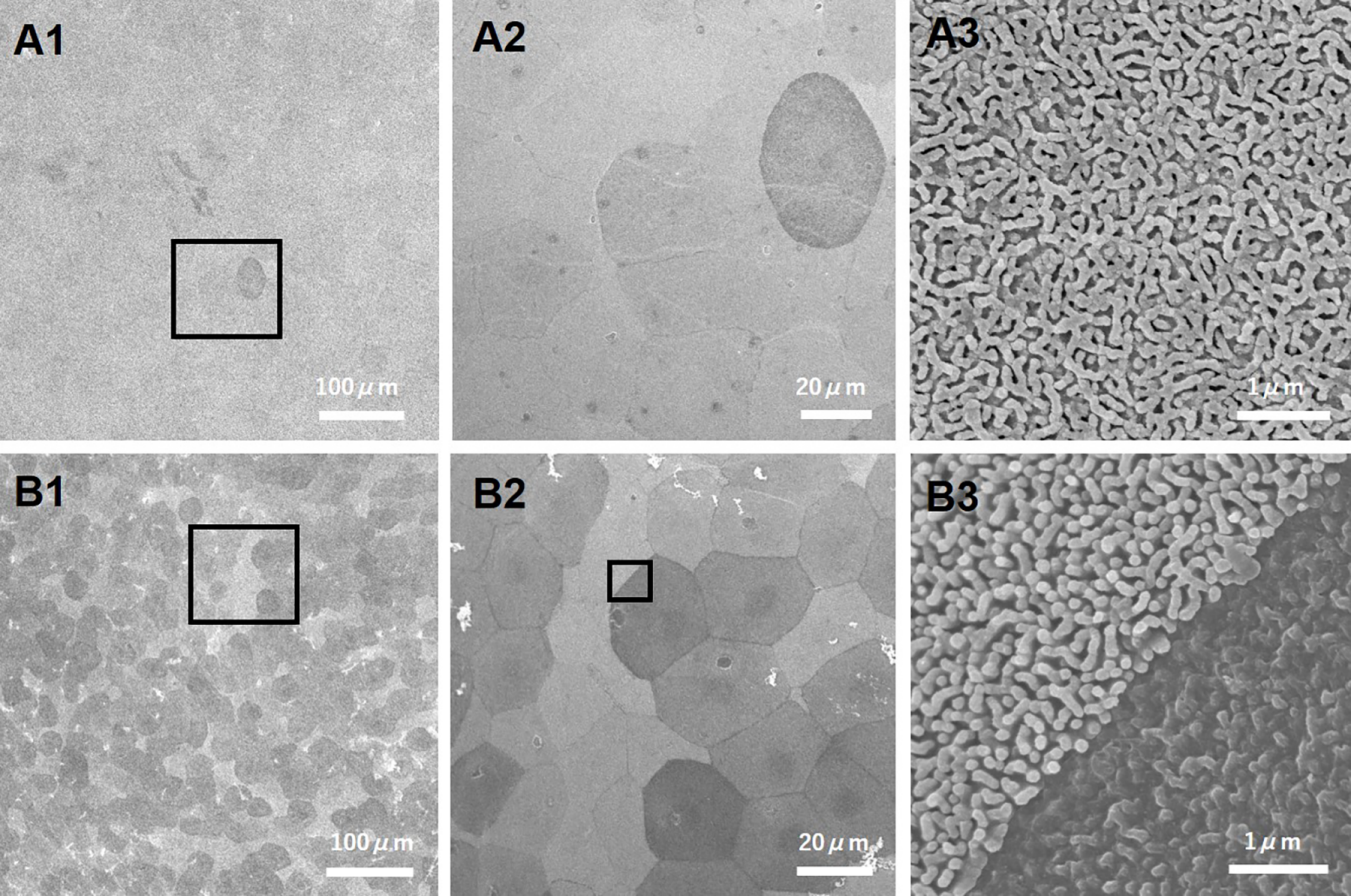
Representative SEM images of the corneal epithelium on day 30. A1-3: untreated group (A1: x100, A2: x400, A3: x15000). The undamaged corneal surface is covered with microvilli. B1-3: Latanoprost/FCTD treated group (B1: x100, B2: x400, B3: x15000). Microvilli were damaged and reduced in the dark cells. A2 and B2 are higher magnification images of the black boxes in A1 and B1, respectively. B3 shows a higher magnification image of the black box in B2.

**Fig 2:**
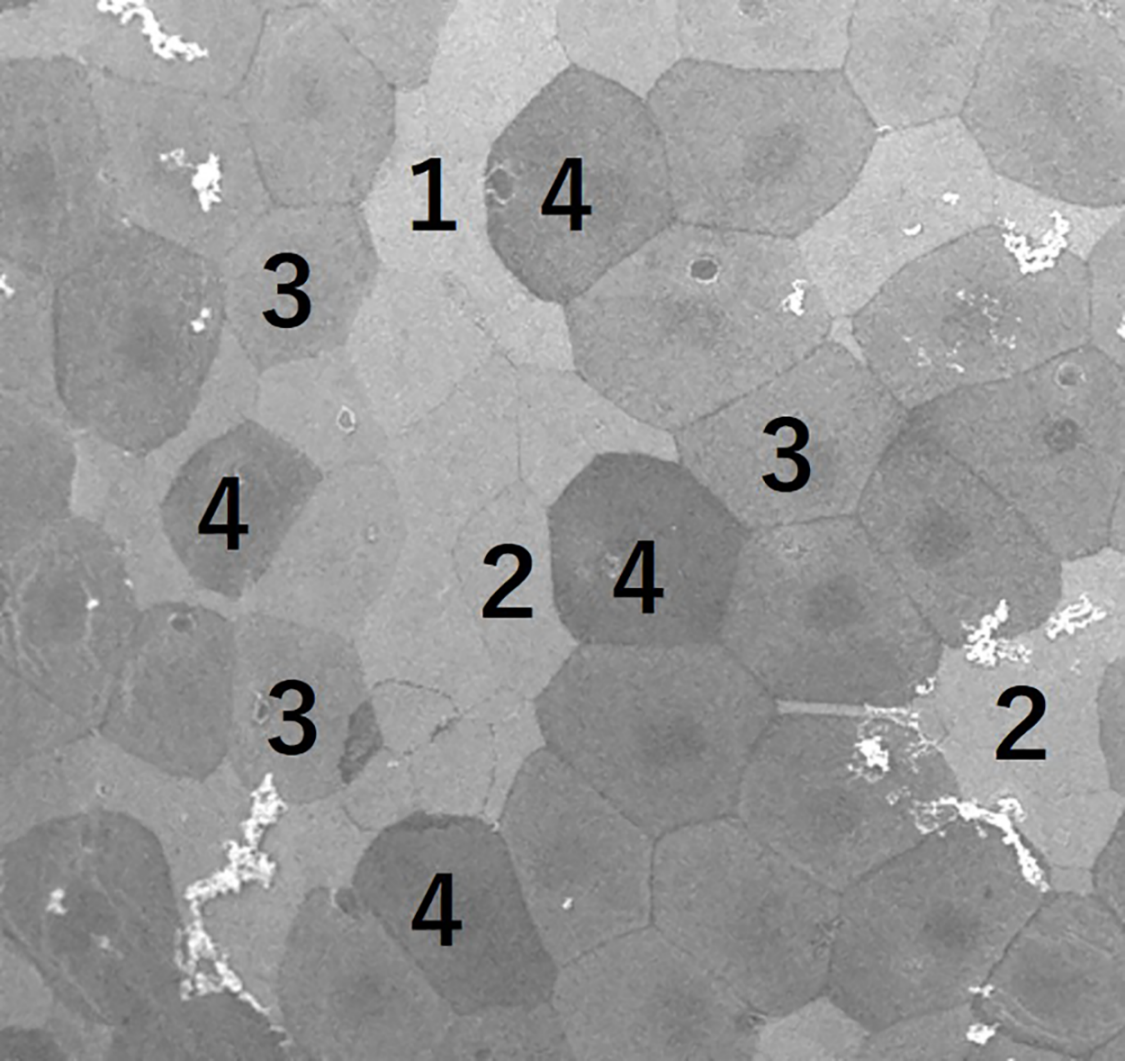
Grading of cell brightness in the corneal epithelium shown at 400x magnification. The corneal epithelial cells were subdivided into 4 intensity levels from lightest to darkest and classified as either bright or dark cells. Cells with an intensity level of 1 or 2 were classified as “bright cells,” and cells with an intensity level of 3 or 4 as “dark cells”.

Corneas were isolated and fixed in 10% formalin and 2% glutaraldehyde. The corneal surface was examined by SEM. Microvilli are a type of microstructure of the corneal epithelium, and when damaged, “dark cells”, which appear to be darkly shaded, can be observed [20, 21] (Fig 1). Dark cells of the corneal epithelium were counted in a masked fashion using images obtained by SEM. First, we obtained SEM images at 400x magnification and cells were subdivided into 4 levels from lightest to darkest (Fig 2). Cells with an intensity level of 1 or 2 were classified as “bright cells,” and cells with an intensity level of 3 or 4 as “dark cells.” Four evaluators used sample images for reference and practiced counting the number of dark cells. Next, to determine if there was a regional difference in the number of glaucoma eyedrop-induced dark cells, we examined five positions within the cornea: the center, and 4 mid-peripheral regions (upper, lower, nasal and temporal) each separated by 2 mm from the center. The right eye was untreated and the left eye was administered latanoprost/FCTD for 30 days. The number of dark cells was counted in three images from each area by two masked evaluators (n = 6). There was a strong correlation between the number of dark cells counted by each evaluator (Pearson correlation coefficient, 0.99; p<0.01).

For the evaluation of glaucoma eyedrops and rebamipide on the ocular surface, the number of dark cells in three images obtained from the center of the cornea was counted by four masked evaluators. There was a strong correlation between the number of dark cells counted by all evaluators (untreated vs. glaucoma eyedrops, intraclass correlation coefficient, 0.914, 95% confidence intervals, 0.720∼0.977; vehicle-treated glaucoma eyedrops vs. rebamipide-treated glaucoma eyedrops: intraclass correlation coefficient, 0.911, 95% confidence intervals, 0.806∼0.962).

### Statistical analysis

Data are presented as the mean ± standard error. The mean score of all evaluators was used. Statistical analyses were performed using SAS software version 9.3 (SAS Institute Inc., NC, USA) and IBM SPSS Statistics version 19 (IBM Corp., NY, USA). A paired *t*-test (two-tailed) was applied for comparisons throughout the study. Inter-rater reliability was evaluated using Pearson’s intraclass correlation coefficient. *P* values of less than 0.05 were considered statistically significant.

## Results

### Glaucoma eyedrop-induced ocular surface disorders

**Fig 3:**
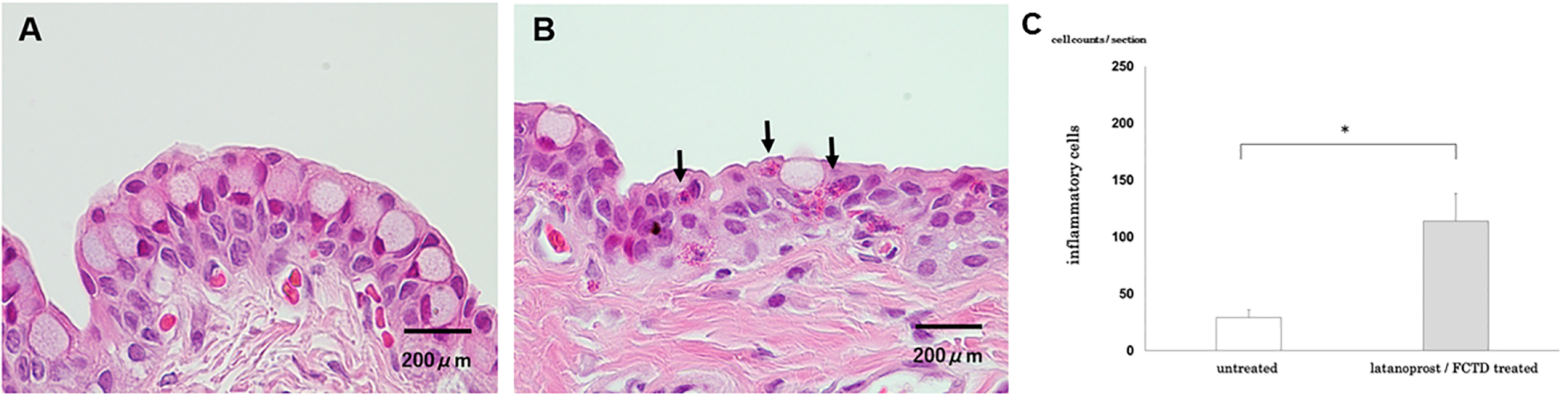
Representative images of Hematoxylin-eosin staining of the conjunctival epithelium on day 30 showing infiltration of inflammatory cells into the conjunctiva due to glaucoma eyedrops. (A) untreated group, (B) latanoprost/FCTD-treated group. Black arrows indicate inflammatory cells. (C) Compared with the untreated group, the number of inflammatory cells in the conjunctival epithelium was significantly increased in the latanoprost/FCTD-treated group (*p<0.05 by a paired two-tailed t-test).

**Fig 4:**
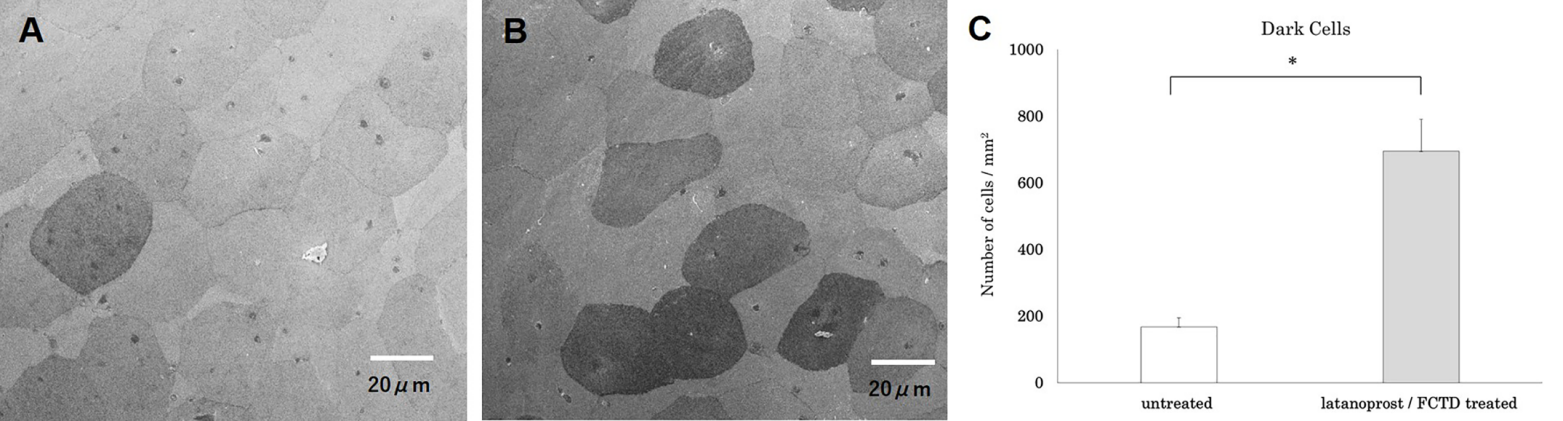
Representative SEM images of corneal epithelial cells on day 30. (A) The untreated group had many bright cells covered with high-density microvilli. (B) The latanoprost/FCTD-treated group showed a large number of dark cells with seriously destroyed microvilli. (C) A significant increase in the number of dark cells was observed in the latanoprost/FCTD-treated group compared to the untreated group (*p<0.05 by a paired two-tailed t-test).

There were no significant differences in fluorescein staining scores between the untreated and latanoprost/FCTD-treated groups on day 30, (untreated group: 0 points, latanoprost/FCTD-treated group, 0.4 ± 0.4 points; p = 0.37). In contrast, significant differences were observed in the rose bengal staining scores (untreated group: 0.4 ± 0.2 points, latanoprost/FCTD treated group: 3.2 ± 0.6 points; p = 0.019). Compared to the untreated group, rose bengal staining scores were increased in the latanoprost/FCTD-treated group on day 30. There was a significant difference (p = 0.039) in the number of inflammatory cells in the conjunctiva among the groups on day 30. Compared with the untreated group, the number of inflammatory cells in the conjunctival epithelium was increased in the latanoprost/FCTD-treated group (untreated group: 29±7 cells/section, latanoprost/FCTD-treated group: 114±24 cell count/section; Fig 3). Based on SEM, there were no regional differences in the number of dark cells in either the untreated or glaucoma eyedrop-treated groups (p>0.05). In the untreated group, a small number of dark cells was present (168±27 cells/mm^2^). In the latanoprost/FCTD-treated group, the cornea showed a large number of dark cells (695±96 cells/mm^2^) which were characterized by seriously destroyed microvilli, diffuse epithelial exfoliation and deformation. The difference observed between these two groups was statistically significant (p = 0.012; Fig 4). For clarification, the number of bright cells was also measured; the number of bright cells was 1002±27 cells/mm^2^ and 681±76 cells/mm^2^ in the untreated and latanoprost/FCTD-treated groups, respectively. The difference in the number of bright cells between these groups was also statistically significant (p = 0.025).

### Evaluation of rebamipide on ocular surface disorders

**Fig 5:**
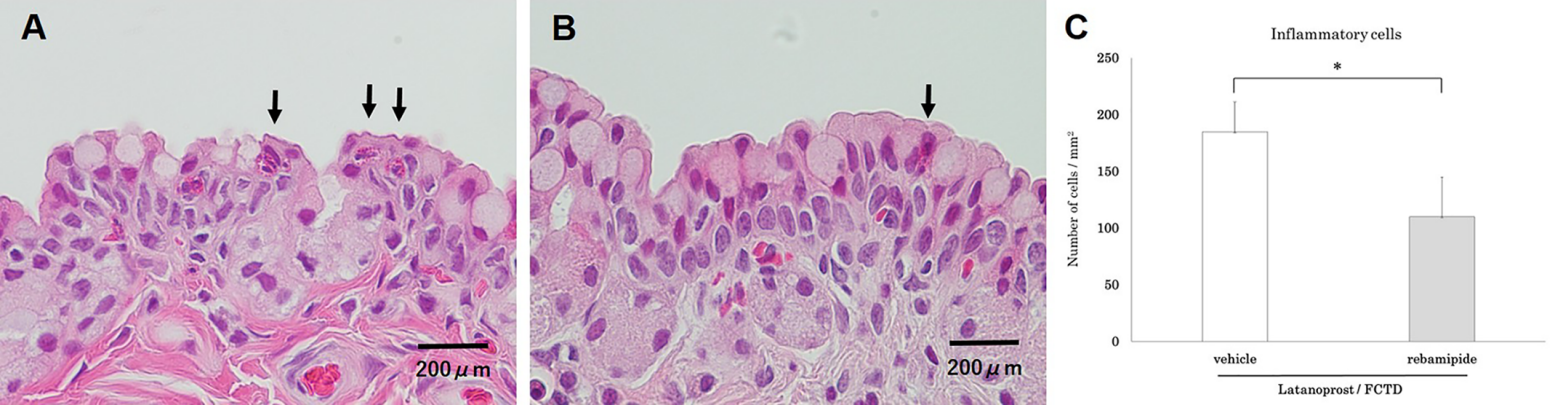
The effect of rebamipide on glaucoma eyedrop-induced inflammatory cell infiltration into the conjunctival epithelium. Representative images of Hematoxylin-eosin staining of the conjunctival epithelium on day 30. (A) Vehicle-treated latanoprost/FCTD group, (B) rebamipide-treated latanoprost/FCTD-treated group. Black arrows indicate inflammatory cells. (C) In the rebamipide-treated latanoprost/FCTD group, conjunctival inflammatory cell infiltration was significantly reduced compared to the vehicle-treated latanoprost/FCTD group (*p<0.05 by a paired two-tailed t-test).

**Fig 6:**
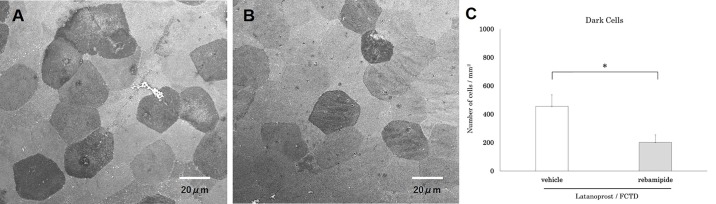
The effect of rebamipide on glaucoma eyedrop-induced dark cells in the corneal epithelium. Representative SEM images of corneal epithelial cells on day 30. (A) The vehicle-treated latanoprost/FCTD group showed a large number of dark cells covered with severely destroyed microvilli. (B) The rebamipide-treated latanoprost/FCTD treated group showed only a moderate increase in the number of dark cells. (C) The number of dark cells in the rebamipide-treated latanoprost/FCTD group was significantly less than in the vehicle-treated latanoprost/FCTD group (*p<0.05 by a paired two-tailed t-test).

In the rebamipide-treated latanoprost/FCTD group, rose bengal staining scores were significantly lower than those in the vehicle-treated latanoprost/FCTD group (rebamipide-treated latanoprost/FCTD group: 1.9±0.4 points, vehicle-treated latanoprost/FCTD group: 3.8±0.4 points; p = 0.003). Furthermore, in the rebamipide-treated latanoprost/FCTD group, conjunctival inflammatory cell infiltration was significantly reduced compared to the vehicle-treated latanoprost/FCTD group (rebamipide-treated latanoprost/FCTD group: 110±35 cells/section, vehicle-treated latanoprost/FCTD group: 185±26 cells/section; p = 0.016, Fig 5). Based on SEM, the cornea of the vehicle-treated latanoprost/FCTD group showed a large number of dark cells covered with seriously destroyed microvilli. The number of dark cells was 456±81 cells/mm^2^. However, in the rebamipide-treated latanoprost/FCTD group, the number of dark cells (202±53 cells/mm^2^) was significantly less than in the vehicle-treated latanoprost/FCTD group (p = 0.011, Fig 6).

## Discussion

Corneal epithelial disorders caused by glaucoma eyedrops are reported to be associated with decreased goblet cell density, indicating a reduction in mucin on the ocular surface [3, 22]. There are two types of mucin on the ocular surface: membranous mucin and secretory mucin. The former is present in the microvilli and the latter is present in the tear film which acts to stabilize the film [23, 24]. By electron microscopy, corneal epithelial cells with microvilli appear as “light cells” and cells without microvilli appear as “dark cells”. Herein we found that rebamipide eyedrops significantly reduced the number of dark cells that had increased in the corneal epithelium by pretreatment with glaucoma eyedrops. This observation indicates an increase in microvilli on the corneal surface by treatment with rebamipide eyedrops, which was confirmed in our subsequent experiments that demonstrated a reduction in the rose bengal score which correlates with damage to the conjunctival mucin layer [25]. Collectively, our current study suggests the potential effects of rebamipide eyedrops on ocular surface protection by demonstrating a reduction in the number of dark corneal epithelial cells (representing cells with defective microvilli) at the cellular level and reduced rose bengal staining.

In this study, we have successfully shown that the numerous inflammatory cells that infiltrate into the conjunctiva due to glaucoma eyedrops were significantly reduced by rebamipide eyedrops, even with simultaneous use of glaucoma eyedrops. These results are consistent with previous findings that rebamipide exerts anti-inflammatory effects by preventing the tumor necrosis factor (TNF)-induced increase in interleukins IL-6/IL-8 [26]. Moreover, rebamipide eyedrops have been shown to exert similar anti-inflammatory effects on the ocular surface membrane [27].

We also observed an increase in the rose bengal scores and a decrease in the number of dark corneal epithelial cells by SEM in the eyes of glaucoma eyedrop-treated rabbits, but no significant changes were noted by examination of fluorescein staining. Moreover, we demonstrated that even after short-term use of glaucoma eyedrops for 30 days, damage to the microstructure of the ocular surface occurs prior to biomicroscopic damage that can be observed with slit lamp examination of fluorescein staining.

In a clinical study, Tokuda et al. reported on the effects of simultaneous use of rebamipide eyedrops and glaucoma eyedrops in glaucoma patients with corneal epithelium disorders [28]. They showed a significant improvement in tear film break-up time and superficial punctate keratopathy within 8 weeks, and improved corneal epithelial barrier function starting at 4 weeks, while IOP remained well controlled.

We should, however, point out a limitation of the current study in that these animal experiments cannot be directly translated to humans. Furthermore, we did not perform quantitative analysis of the goblet cells nor mucin levels. Lastly, in this study the eyedrops were only administered for 30 days, whereas patients with glaucoma typically use eyedrops for an extended period of time.

In conclusion, results from our *in vivo* rabbit study found that glaucoma eyedrops induced corneal epithelium disorders at the cellular level during short term use, but that simultaneous use of rebamipide has the potential to protect and repair the ocular surface. Further clinical evaluation over a longer period of time with a large number of patients will be required to fully elucidate the clinical utility of rebamipide eyedrops for patients with glaucoma.

## Supporting information

S1 Fig Influence of latanoprost/FCTD treatment on the number of bright cells in the rabbit corneal epithelium on day 30. A significant decrease in the number of bright cells was observed in the latanoprost/FCTD-treated group compared to the untreated group (*p = 0.025 by a paired two-tailed t-test). Data are presented as the mean ± standard error (n = 5).
(TIF)
