# Supplementary figures and images for: Rebamipide protects against glaucoma eyedrop-induced ocular surface disorders in rabbits
Source: PLoS One. 2017 Oct 19;12(10):e0186714. doi: 10.1371/journal.pone.0186714 (PMC5648230; doi:10.1371/journal.pone.0186714)

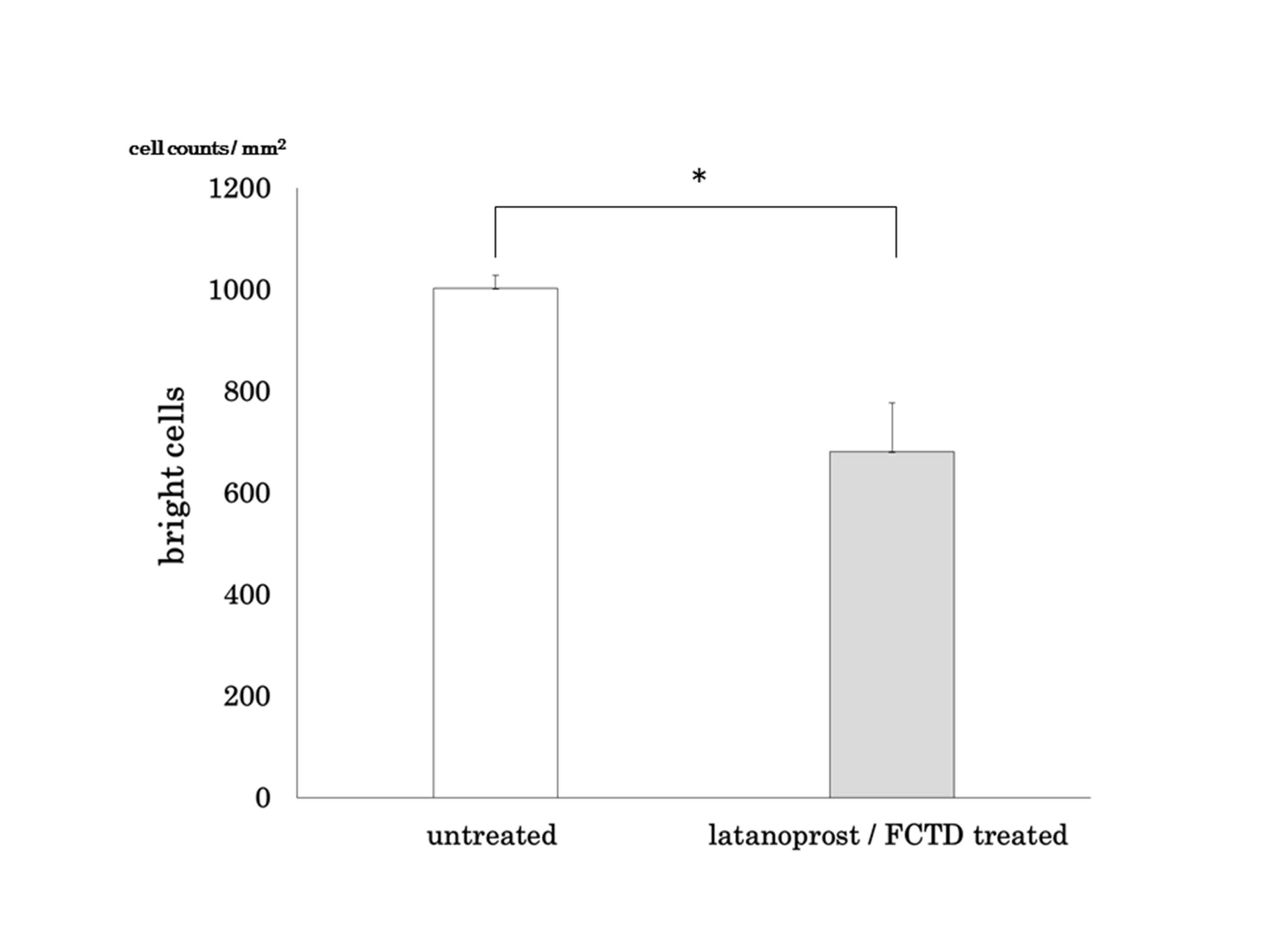

Supplement: S1 Fig — A significant decrease in the number of bright cells was observed in the latanoprost/FCTD-treated group compared to the untreated group (*p = 0.025 by a paired two-tailed t-test). Data are presented as the mean ± standard error (n = 5). (TIF) [file pone.0186714.s001.tif]
